# Genome-Wide Screen of miRNAs and Targeting mRNAs Reveals the Negatively Regulatory Effect of miR-130b-3p on PTEN by PI3K and Integrin β1 Signaling Pathways in Bladder Carcinoma

**DOI:** 10.3390/ijms18010078

**Published:** 2016-12-31

**Authors:** Mengxin Lv, Zhenyu Zhong, Hong Chi, Mengge Huang, Rong Jiang, Junxia Chen

**Affiliations:** 1 Department of Cell Biology and Genetics, Chongqing Medical University, Chongqing 400016, China; 15123824592@163.com (M.L.); 15310432691@163.com (H.C.); 2 The First Clinical College, Chongqing Medical University, Chongqing 400016, China; mylifeemail@163.com; 3 College of Clinical Medicine, Southwest Medical University, Luzhou 646000, China; 18983004784@163.com; 4 Laboratory of Stem Cells and Tissue Engineering, Chongqing Medical University, Chongqing 400016, China; 13150535015@163.com

**Keywords:** miRNA, integrin β1, miR-130b, PTEN, invasion, bladder cancer

## Abstract

miRNAs have emerged as promising markers for tumors. However, the underlying mechanism of specific miRNAs in bladder cancer (BC) remains largely unknown. Here, a comprehensive miRNA/mRNA expression profile was executed by microarray assay for four pairs of bladder carcinoma and para-carcinoma tissues from patients with grade 2 (G2) T2. A total of 99 miRNAs and 4416 mRNAs were discovered to be significantly differentially expressed in BC tissues compared with controls. Five microRNAs and two mRNAs were validated by qRT-PCR in 30 pairs of samples, including G1–G3/T1–T4. Subsequently, we constructed a network with the five miRNAs-target mRNAs; gene ontology (GO) and Kyoto Encyclopedia of Genes and Genomes (KEGG) enrichment analyses were utilized to recognize the functions and associated pathways. Moreover, we further found that miR-130b-3p was significantly up-regulated and negatively correlated with phosphatase and tensin homolog (PTEN) expression in bladder cancer tissues. Next, we demonstrated that miR-130b-3p might target PTEN through bioinformatics and dual-luciferase reporter assay. Finally, we showed that miR-130b-3p could down-regulate PTEN expression, which promoted proliferation, migration, invasion and rearranged cytoskeleton through the activation of the PI3K and integrin β1 signaling pathway in bladder cancer cells. Inversely, miR-130b-3p inhibitors induced apoptosis. Taken together, this research investigated, for the first time, miR-130b-3p by an incorporated analysis of microRNA/mRNA expressions of a genome-wide screen in BC. Our findings suggest that the miR-130b-3p/PTEN/integrin β1 axis could play a critical role in the progression and development of BC and that miR-130b-3p might be a valuable clinical marker and therapeutical target for BC patients.

## 1. Introduction

Worldwide, approximately 440,000 new bladder tumors are diagnosed and 150,000 patients died from them yearly. A high recurrence rate is one representative characteristic of bladder cancer. Nearly 15%–40% of the recurrent bladder cancer (BC) patients exhibit invasive characteristics and distant metastasis. Limited therapeutic options for invasive bladder cancer have resulted in a median survival of 15 months for patients with metastatic disease. At present, there are not any molecular targets to be approved for therapy of BC. Therefore, this highlights the urgent need for novel therapeutic targets with improved clinical outcomes [1,2].

It is well known that microRNAs are a class of small noncoding RNAs with a length of 17–24 nucleotides. MicroRNAs could inhibit protein translation or degrade target mRNAs to regulate gene expression at post-transcriptional level. Accumulating evidence demonstrates that microRNAs participate in various biological processes and exert momentous effects in the pathogenesis and development of BC [3]. Genome-wide prediction suggests that more than 60% of protein coding genes can be regulated by miRNAs. The specific dysregulation of miRNAs is associated with different cancers. Emerging evidence has reported that miRNAs are remarkably aberrant in tumors, and may be implicated in tumorigenesis and the development of BC [4,5]. However, specific biomarkers of BC have not been found; in addition, due to their inherent nature, miRNAs appear to be highly stable and provide more accurate prediction for clinical specimens. This shows the prospective in applying specific miRNAs for diagnosis and therapeutics of cancer [6]. Several researches have shown the role of the miR-130 family in cancer progression [7,8,9], but comprehensive analysis of the miRNA/mRNA expression profile to reveal the effects of the miR-130-3p on tumor progression and development, including bladder cancer, has not been reported so far.

Previous investigations showed that advanced stage bladder cancer (≥pT2) is associated with the mutation of tumor suppressor gene PTEN, which could play a negative regulatory role in the PI3K/Akt signaling pathway [10,11]. Furthermore, Calderaro et al. reported that 129 bladder cancer samples revealed an activation of the PI3K/AKT pathway in the whole spectrum, including various stages of bladder cancer. Integrated analysis suggests that the PI3K/Akt pathway is one of three frequently dysregulated pathways in BC [12,13]. Also, it has been demonstrated that PTEN could directly dephosphorylate protein tyrosine kinase 2 (FAK) tyrosine phosphate and inhibit integrin-mediated cell migration, invasion, and cytoskeleton arrangement [14]. The integrin β1 (ITGB1) family mainly transduces signals from the extracellular matrix to modulate growth and differentiation, and has been implicated in cell proliferation, adhesion and metastasis in a wide variety of human cancers, including breast, colon and ovary [15]. However, whether miR-130b modulates the activity of the integrin β1 signaling pathway via PTEN in BC has not been elucidated.

Here, we performed a comprehensive analysis of miRNA/mRNA expression profiles to probe dysregulation of miRNAs/mRNAs and molecular mechanisms underlying the development of BC on the base of a microarray from four pairs of bladder carcinoma and para-carcinoma tissues from patients with grade 2 (G2) T2. A miRNA-target gene regulatory network was constructed. Bioinformatics gene ontology and pathway analyses were executed. For the first time, we integrated miRNA/mRNA expression profiling to screen target molecules in BC. We discovered that up-regulating miR-130b-3p was negatively associated with PTEN and stanniocalcin 1 (STC1) in clinical bladder cancer specimens. Moreover, the results further demonstrated that the miR-130b-3p facilitated bladder cancer cell proliferation, migration and invasion targeting PTEN through the PI3K and integrin β1 signaling pathway. Our study also revealed that the expressions of a large number of miRNAs were altered in BC compared with normal tissues, especially suggesting that the miR-130b-3p/PTEN/integrin β1 axis could potentially serve as a clinical marker for the diagnosis and therapy of BC.

## 2. Results

### 2.1. Differentially Expressed miRNA and mRNA Profiles Are Screened by Microarray

**Figure 1:**
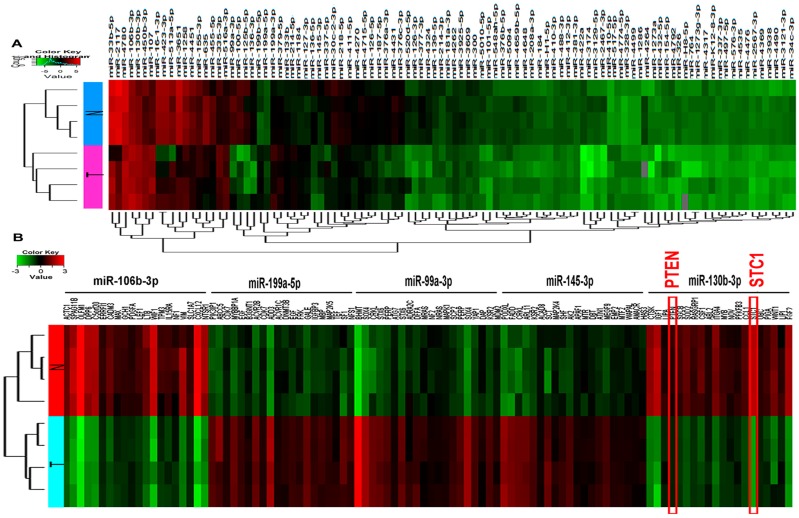
Heat maps show expression profiles of miRNAs and co-expressed target mRNAs. Each row represents a sample and each column represents a miRNAs or mRNA. The red strip represents high relative expression and the green strip represents low relative expression. T represents the bladder cancer group, and N represents the normal control group. Each group contains four different samples. (**A**) Heat map of differentially expressed miRNAs (fold change ≥ 1.5 and *p*-value < 0.05); and (**B**) Heat map of co-expressed target mRNAs from the two most up- and the three most down-regulated miRNAs.

To evaluate the relationships among the samples, hierarchical clustering analysis was applied to group the samples based on the expression levels. The relationships among the miRNA expression modes between specimens are represented by a dendrogram. The miRNA expression profile using the microarray contained a total of 99 miRNAs (18 up-regulated miRNAs and 81 down-regulated miRNAs) that were significantly differentially expressed in the BC tissue samples compared with para-carcinoma tissues (fold change ≥1.5). Down-regulated miRNAs were more frequent than up-regulated miRNAs in our microarray signature (Figure 1A). A total of 4416 mRNAs were detected to be differentially expressed with fold-change ≥2.0 and *p* < 0.05. Hierarchical clustering for mRNAs showed co-expressing target genes of the two most up- and three most down-regulated miRNAs according to their expression abundances (hsa-miR-199a-5p, hsa-miR-145-3p, hsa-miR-130b-3p, hsa-miR-106b-3p and hsa-miR-99a-3p) (Figure 1B, Table 1 and Table 2).

### 2.2. The Regulatory Network of miRNAs and Target Genes Are Constructed

**Figure 2:**
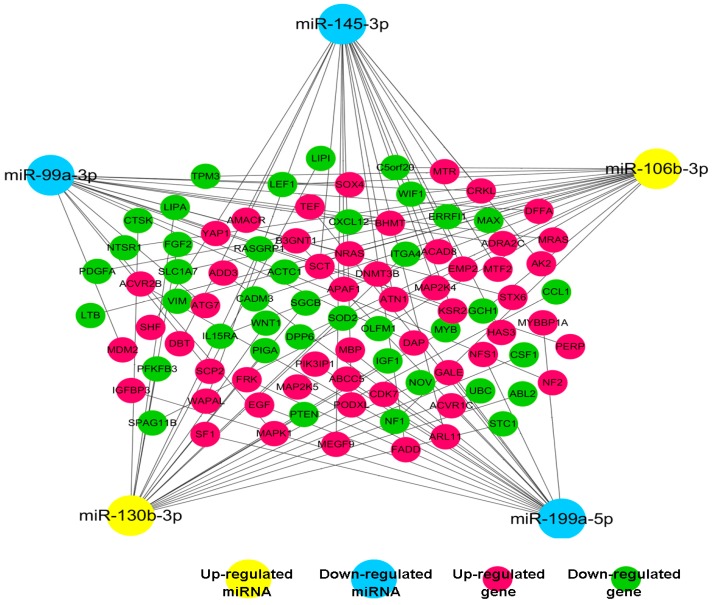
The co-expression network is constructed with representative miRNAs and their target genes. Solid lines mean negative correlations between five representative miRNAs and their targets (the absolute value of Pearson correlation coefficient (PCC) ≥ 0.90, *p*-value < 0.01 and false positive rate (FDR) < 0.01).

Next, to determine the interactions between miRNAs and their target mRNAs, a miRNA–mRNA interacting network of the five confirmed miRNAs and their co-expressing targets was produced using cytoscape software. These targets were obtained utilizing the Mir-Walk database. The result showed that five miRNAs and 50 genes constituted miRNA-target mRNA pairs with negative correlation, some candidate targets are connected by only one miRNA, but many can be regulated by multiple miRNAs to affect their expression levels (Figure 2).

### 2.3. Differently Expressed miRNAs Are Identified

**Figure 3:**
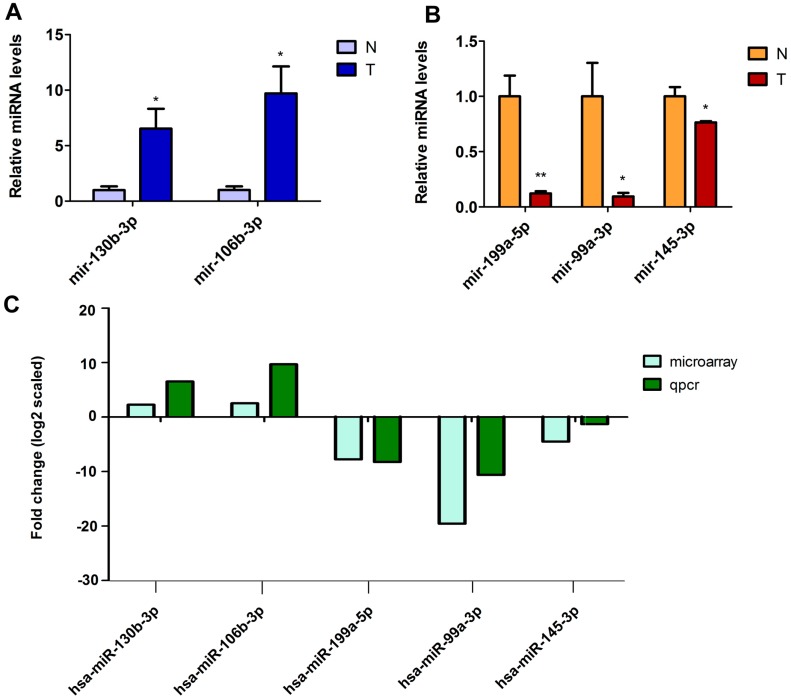
The differentially expressed miRNAs are validated in bladder carcinoma by quantitative RT-PCR. (**A**,**B**) The relative expression levels of the five miRNAs are shown in thirty pairs of tumor tissues (T) and normal tissues (N). Data are shown as mean ± SEM. * *p* < 0.05, ** *p* < 0.01, *n =* 30; and (**C**) The comparison between qPCR results and microarray data. The heights of the columns represent the fold changes (log2 transformed) computed from qPCR and microarray data respectively.

According to the significance of fold change, five microRNAs among these were picked to verify the microarray data using qRT-PCR in 30 BC samples. Details of the clinicopathological data are shown in Supplementary Table S1. The results displayed that the expressions of mir-130b-3p and mir-106b-3p were upregulated (Figure 3A), whereas mir-99a-3p, mir-199a-5p and mir-145-3p were downregulated (Figure 3B), which agree with the microarray data. Therefore, the qRT-PCR data verified the accuracy of microarray results (Figure 3C). The data supply evidence that these miRNAs could be involved in the tumor development of bladder cancer.

### 2.4. Kyoto Encyclopedia of Genes and Genomes Pathway and Gene Ontology Terms Classifications Are Analyzed

**Figure 4:**
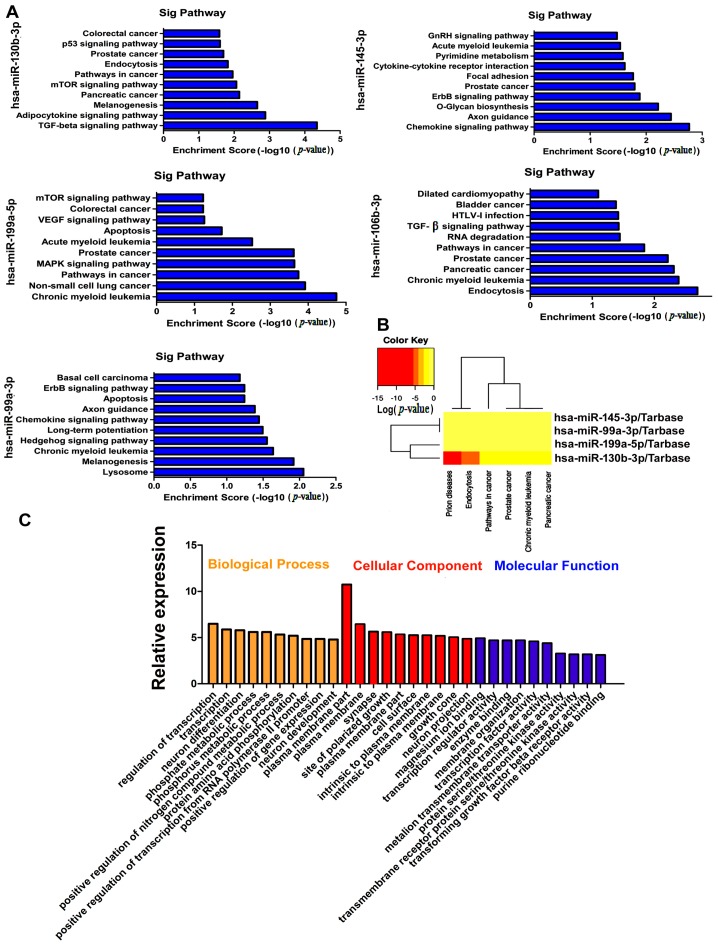
Pathways of target genes for the five representative miRNAs are analyzed. (**A**) KEGG pathway enrichment analysis with a top ten Enrichment score; (**B**) Hierarchical clustering of miRNA related pathways. The heatmap of the miRNAs merged pathway reveals significance by *p*-value (log scaled). Red represents high significance; and (**C**) DAVID analysis of target genes of miR-130b-3p.

Based on the latest KEGG database, we analyzed the biological pathways with significant enrichment of differentially co-expressing target mRNAs of the five miRNAs. The data showed that up-regulated transcripts and down-regulated transcripts corresponded to 10 pathways. The colorectal cancer, p53 signaling pathway and pathways in cancer were the top pathways enriched among protein-coding target genes of hsa-miR-130b-3p, whereas, the chronic myeloid leukemia, non-small cell lung cancer and pathways in cancer were the top pathways enriched among target genes of hsa-miR-99a-3p (Figure 4A). The data suggest that these pathways might have significant implications on the tumorigenesis and development of bladder cancer. Then, DIANA-miRPath analysis revealed that hsa-miR-130b-3p, hsa-miR-199a-5p, hsa-miR-145-3p and hsa-miR-99a-3p were associated with pathways in cancer. It has been indicated that miRNA-130b has the highest correlation with cancer-associated signal pathways in the four miRNAs (Figure 4B). Next, we would focus on miRNA-130b for further research. The GO categories are functional-analysis-associated differentially expressed mRNAs including cellular components, biological processes and molecular functions. GO enrichment analysis of differentially expressed target mRNAs for mir-130b-3p might supply comprehension for the function of mir-130b-3p. The data suggested that mir-130b-3p is largely associated with biological processes, and that molecular functions were the regulation of transcription, transcription regulator activity, phosphate metabolic process, protein amino acid phosphorylation and protein serine/threonine kinase activity. Importantly, these GO terms are critical for controlling carcinogenesis and the development of tumors (Figure 4C).

### 2.5. Target Gene PTEN of miR-130b-3p Is Predicted and Verified

**Figure 5:**
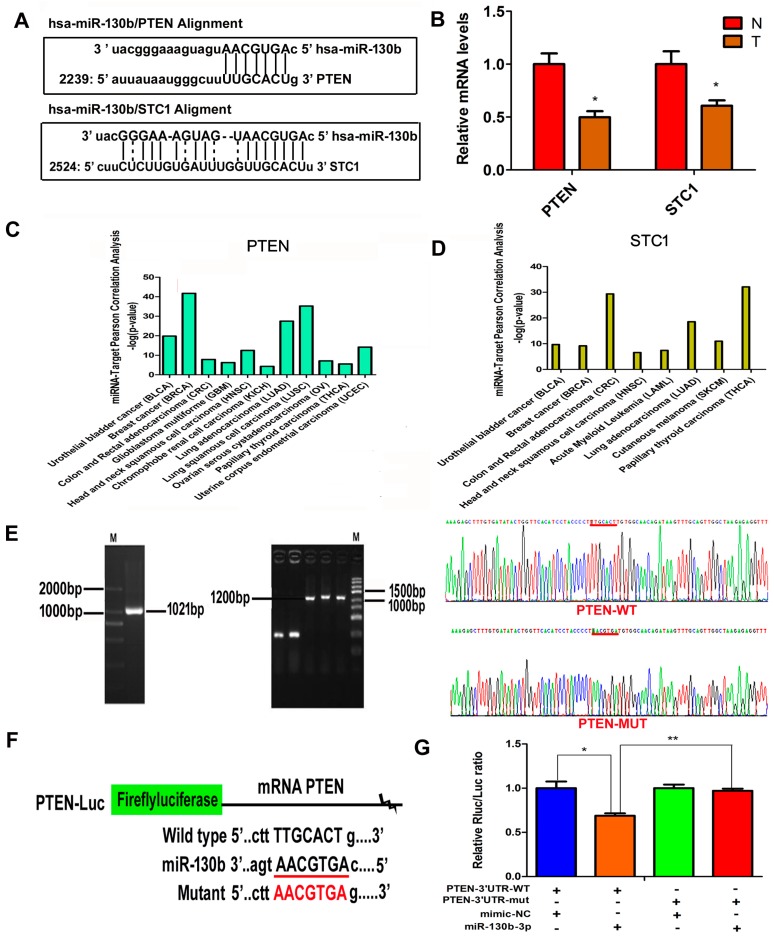
Targets of miR-130b-3p are identified. (**A**) The putative binding sites between miR-130b-3p and PTEN 3′-UTR or stanniocalcin 1 (STC1) 3′-UTR; (**B**) The relative expression levels of PTEN and STC1 in thirty pairs of tumor tissues (T) and normal tissues (N). Data are shown as mean ± SEM. * *p* < 0.05, *n =* 30; (**C**,**D**) miR-130b/target PTEN or STC1-cancers Pearson correlation analysis according to starbase function prediction; and (**E**–**G**) Luciferase activity in 293T cells co-transfected miR-130b-3p with luciferase reporters containing PTEN 3′-UTR or mutant. Data are presented as the relative ratio of Renilla luciferase activity to fireflyluciferase activity. *n* = 3, mean ± SD. * *p* < 0.05, ** *p* < 0.01. The red lines in the Figure 5E,F are the binding site and mutation site of PTEN binding to miR-130b-3p. The red character in the Figure 5F is the mutation sequence.

Bioinformatic prediction (TargetScan and miRDB) indicated that the tumor suppressor genes PTEN and STC1 could be potential targets of miR-130b (Figure 5A). Two target mRNAs of mir-130b were chosen for confirmation of the microarray assay with qRT-PCR in 30 pairs of samples. The qRT-PCR result displayed that the expressions of two target mRNAs—PTEN and STC1—were both downregulated consistently in BC compared with adjacent non-tumorous tissues, respectively. The expression tendency of these mRNA targets was opposite to the expression of their corresponding miRNAs (Figure 5B). These findings suggest that the mRNAs could be implicated in pathogenesis of bladder cancer. Next, the disease association analysis for target genes PTEN and STC1 were performed using Starbase software (starbase Function Prediction). The results showed that the two target genes were both associated with bladder cancer, and PTEN showed a higher correlation than STC1 (Figure 5C,D). Then, to verify miR-130b-3p directly targeting the 3′-untranslated Regions (3′-UTR) of PTEN mRNA, a dual-luciferase reporter assay was executed. The luciferase activity of 293T cells transfected with miR-130b mimics was decreased by about 64% compared with the control, which suggests that PTEN is a target gene of miR-130b (Figure 5E–G).

### 2.6. The miR-130b-3p Affects Proliferation and Apoptosis of Bladder Cancer Cells

**Figure 6:**
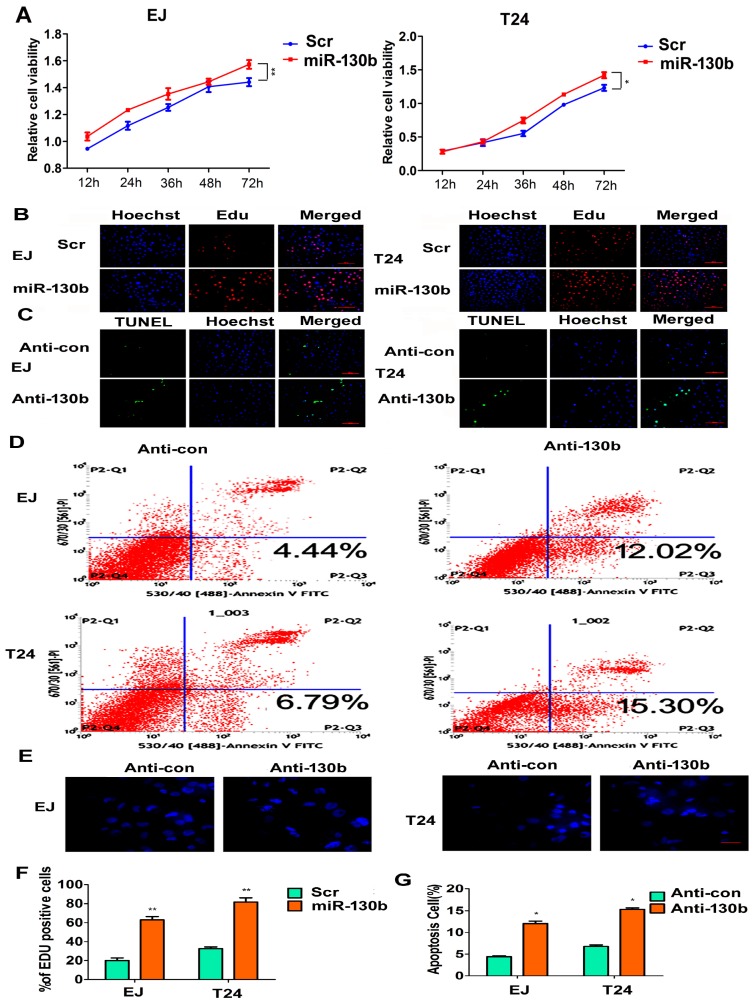
miR-130b-3p affects cell proliferation and apoptosis. (**A**) CCK8 analysis of EJ or T24 cells transfected with miR-130b-3p mimics or the scramble control; * *p* < 0.05, ** *p* < 0.01; (**B**) Representative images of the Edu assay of EJ or T24 cells transfected with miR-130b-3p mimics or the scramble control. Hoechst stains the nucleus. Scale bar = 100 μm; The blue spots are the nuclear, the red spots are the proliferative cells; (**C**) Representative photographs of TUNEL staining of EJ or T24 cells transfected with inh-130b or the scramble control. Hoechst stains the nucleus. Scale bar = 100 μm; The blue spots are the nuclear, the green spots are the apoptotic cells; (**D**) Flow cytometry analysis with Annexin V-PI staining. The percentage of apoptotic cells in EJ or T24 groups transfected with anti-130b or anti-con, respectively; (**E**) Representative nuclei images of Hoechst staining. Scale bar = 50 μm; (**F**) Quantitative Edu assay data in five fields. Mean ± SD. ** *p* < 0.01; and (**G**) Quantitative Tunel assay data in five fields. Mean ± SD. * *p* < 0.05.

CCK8 (Cell Count Kit-8) and Edu (5′-ethynyl-2′-deoxyuridine) assays were executed to determine the effects of miR-130b-3p on the proliferative ability of EJ and T24 bladder cancer cells. The data curves showed that the cell viability with transfection of the miR-130b mimics was increased compared with the scramble control groups (Figure 6A). Similar results were obtained from the Edu assay (Figure 6B,F). Flow cytometry and a terminal-deoxynucleoitidyl Transferase Mediated Nick End Labeling (TUNEL) analysis were implemented to detect the roles of anti-130b on apoptosis in vitro. The result showed that TUNEL-positive cells were significantly enhanced in the anti-130b groups, compared with anti-con groups (Figure 6C,G). In the flow cytometry assay shown in Figure 6D, about 12.02% and 15.3% of cells presented apoptosis in the anti-130b groups, while approximately only 4.44% and 6.79% of cells presented apoptosis in anti-con groups. The Hoechst 33342 staining revealed that anti-130b cells displayed a representative morphological apoptosis characteristic, such as the nuclear fragment, the apoptotic body and chromatinic condensation, while, the control cells did not indicate apoptosis traits (Figure 6E).

### 2.7. The miR-130b-3p Promotes Invasion, Migration and Rearranges Cytoskeleton of Bladder Cancer Cells

**Figure 7:**
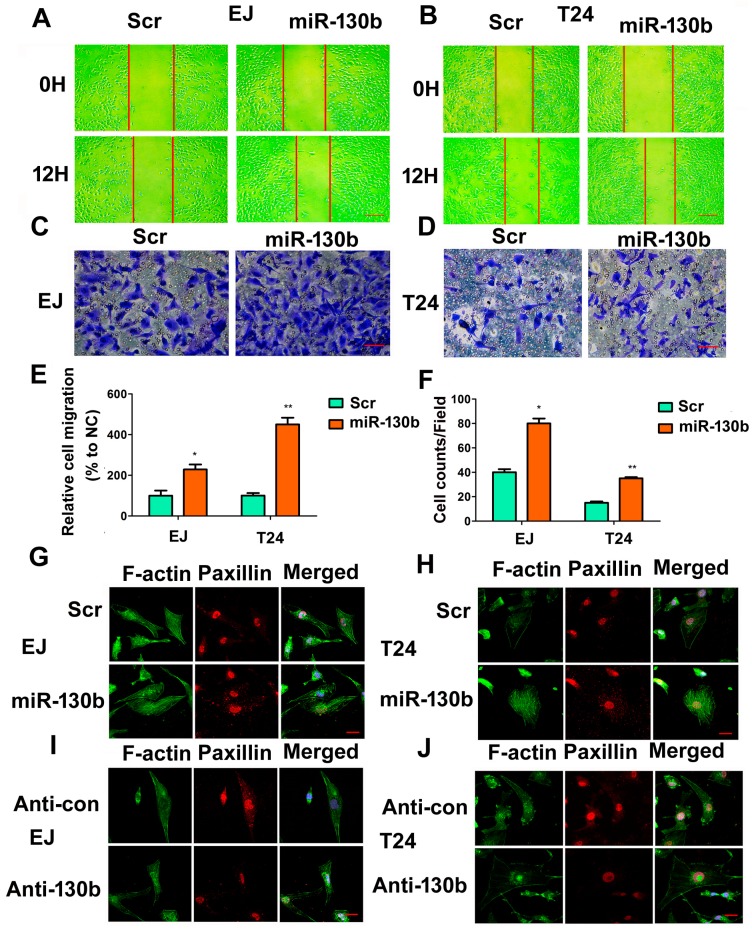
miR-130b-3p promotes cell migration and invasion capability. (**A**,**B**) Wound healing assay of EJ and T24 cells transfected with miR-130b-3p mimics or the scramble control at 0 and 12 h respectively. Scale bar = 200 μm; The red lines are the scratch position; (**C**,**D**) Transwell assay. Scale bar = 100 μm; (**E**) Quantitative wound assay data. Data are the mean ± SD of wound width in three experiments. * *p* < 0.05, ** *p* < 0.01; (**F**) Quantitative transwell assay data. Data are the mean ± SD of invaded cell counts in five fields. * *p* < 0.05, ** *p* < 0.01; and (**G**–**J**) Phalloidine-fluorescein isothiocyanate (FITC) staining of microfilament (**green**) and skeleton molecule paxillin (**red**) under confocal laser scanning microscopy. Scale bar = 20 μm.

The migration and invasion of cancer cells are one of the leading reasons of metastasis. Subsequently, we detected the effects of miR-130b-3p on the ability of migration and invasion in EJ and T24 cells. Up-regulating miR-130b-3p significantly facilitated migration and invasion of cells (Figure 7A–F). F-actin and paxillin were stained with fluorescent phalloidin and Immunofluorescence. The miR-130b antagomirs markedly suppressed stress fiber formation and the expression of paxillin in cells under fluorescent microscopy. Instead, miR-130b-3p resulted in more abundant bundles of actin filaments and a brighter fluorescent signal of paxillin (Figure 7G–J).

### 2.8. miR-130b-3p Regulates PI3K and Integrin β1/FAK Signaling Pathways Targeting PTEN

**Figure 8:**
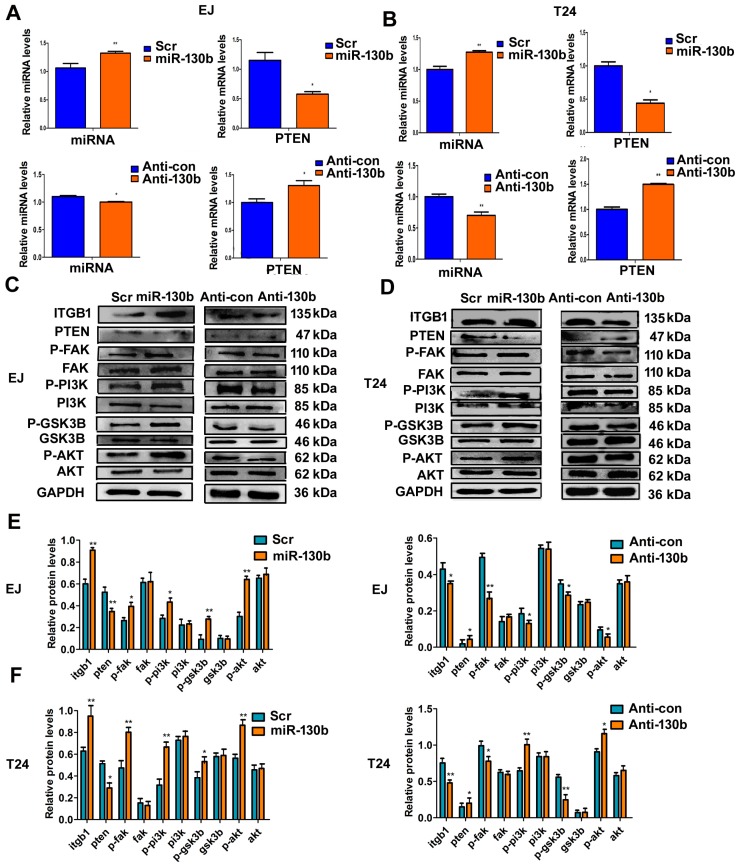
miR-130b-3p affects the expression of PTEN and modulates PI3K/AKT and integrin β1/FAK signaling pathways. (**A**,**B**) The relative expression levels of miRNA miR-130b and mRNA PTEN in cells transfected with miR-130b mimics, the scramble control, anti-130b or anti-con by qRT-PCR; (**C**,**D**) Western blot analysis; and (**E**,**F**) Quantitative analysis of relative protein levels. Data are expressed as mean ± SD (*n* = 3), * *p* < 0.05, ** *p* < 0.01.

To illuminate the potential molecular mechanism of miR-130b, EJ and T24 cells were transfected with miR-130b-3p mimics, the scramble control, anti-130b or anti-con, respectively. qRT-PCR results revealed that levels of PTEN were negatively regulated by miR-130b (Figure 8A,B). Subsequently, we explored whether miR-130b could affect p-PI3K, p-Akt, p-FAK and integrin β1 protein levels by targeting PTEN. Western blotting showed that PTEN is the major negative regulator of the PI3K and integrin β1/FAK pathways. The up-regulating miR-130b increased the expressions of p-PI3K, p-Akt, p-FAK and integrin β1. Conversely, these protein levels were decreased when the cells were transfected with anti-130b (Figure 8C–F).

### 2.9. miR-130b-3p Suppresses the Expression Target Gene PTEN

**Figure 9:**
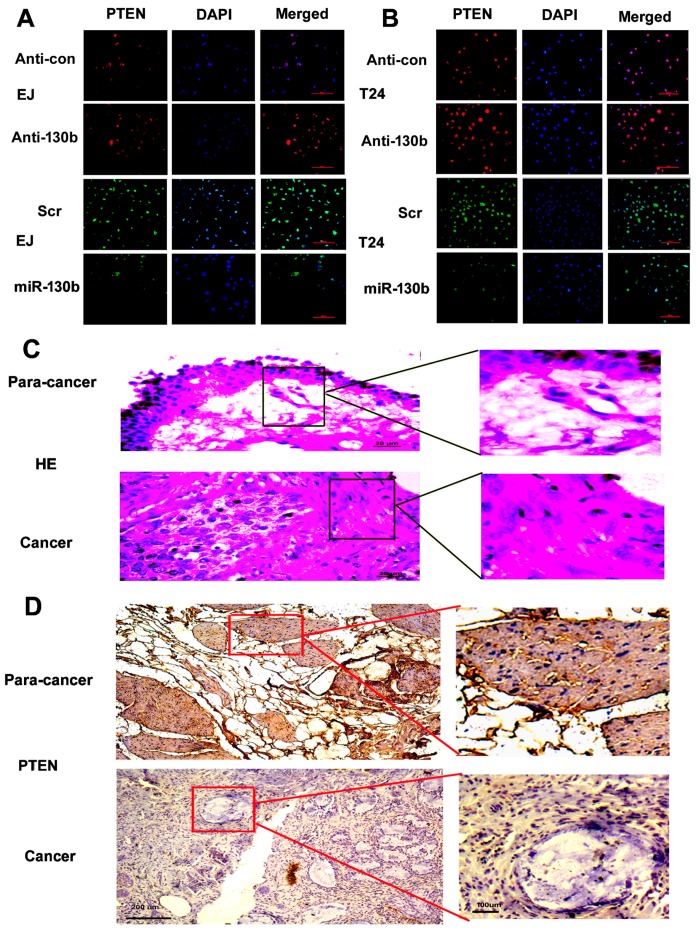
miR-130b-3p regulates the expressions of PTEN in cells and the expressions of PTEN are determined in human tissues. (**A**,**B**) Immunofluorescence analysis of PTEN in EJ and T24 cells transfected with miR-130b mimics, the scramble control, anti-130b or anti-con, respectively. Scale bar = 100 μm; (**C**) Representative photographs of hematoxylin-eosin (HE) staining of bladder carcinoma and para-carcinoma tissues. Scale bar = 20 μm; and (**D**) Representative images of immunohistochemical staining of tissues with antibody of PTEN. Scale bar = 200 μm (**left**), 100 μm (**right**).

Immunofluorescence detection revealed that anti-130b resulted in a much higher PTEN expression than the anti-con group, whereas, a weaker fluorescent signal of PTEN was observed in miR-130b-3p cells groups compared with the scramble control groups in vitro (Figure 9A,B). Immunohistochemical assays of PTEN were further determined in the human bladder tissue. Cancer tissues displayed much weaker positive staining of PTEN expression compared with adjacent non-tumor tissues in vivo (Figure 9C,D). The results are consistent with Western blot and the findings agree with the prediction that miR-130b-3p could suppress the expression PTEN by binding to the 3′-UTR of PTEN.

## 3. Discussion

According to data, there is no molecular target to be approved for the treatment of urothelial carcinoma of the bladder [16]. As far as we know, a genome-wide screen of miRNAs to elucidate the function of specific miRNAs by integrated miRNA and mRNA expression profiles in BC has not been reported. Here, we integrated mRNA/miRNA expression data on the base of a microarray to find a new regulatory relation between miRNAs and mRNAs with miRNA target predictions by the miRWalk database. Then, a regulatory network of miRNA/target mRNA pairs was built. Five miRNAs and 50 genes formed the miRNA-target mRNA pairs with a negatively correlated expression. In this network, hsa-miR-130b-3p was one of the most represented connectivities, while PTEN was revealed to be the mRNA with the most links, suggesting that hsa-miR-130b-3p and PTEN might be implicated in the oncogenesis of BC. Next, five selected miRNAs (two of the most up- and three of the most down-regulated miRNAs) and two mRNAs (miR-130b’s targeting gene) were validated on 30 samples using qRT-PCR. MicroRNA is an attractive potential diagnostic biomarker due to its high stability and easy quantification. Epigenetic and microRNA regulation are associated with carcinogenesis and the development of bladder cancer. The global expression profile of miRNAs supplies an effective approach for actual use of miRNAs; a single miRNA or panel of several miRNAs might elevate the accuracy of risk stratification and pathological diagnosis for BC [17].

In addition, function annotation for miRNA target mRNAs of the co-expression with miRNAs was executed to evaluate the biological functions of differentially expressed miRNAs in BC. The predicted targets were found to be significantly dysregulated in regulation of transcription, protein serine/threonine kinase activity and positive regulation of gene expression of GO terms, which are closely relevant to the biological character of cancer cells [18,19]. KEGG pathway analysis indicated that pathways in cancer, the mammalian target of rapamycin (mTOR) signaling pathway and the p53 signaling pathway were significantly enriched with differentially expressed genes including *IGF1*, *WNT1*, *ADAM12*, *SP1* and *ACVR1*, etc. These genes could function as oncogenes and tumor suppressor genes or were implicated in the regulation of oncogenes and tumor suppressor genes to correlate with the tumorigenesis and the development of bladder cancer [20,21,22].

Recently, several reports have shown the impact of the miR-130 family on cancer progression; miR-130b is significantly dysregulated in a wide variety of tumors, such as glioma, gastric cancer, renal cell cancer and endometrial cancer [23,24,25]. However, the comprehensive effect of the miR-130b-3p molecules on tumor progression, including bladder cancer has not been well understood. Our miRNA and mRNA microarray data and qRT-PCR analysis verified that miR-130b-3p was up-regulated and PTEN was down-regulated in bladder cancer tissues. Using silico analyses, the tumor suppressor gene, PTEN, was predicted as a significant target of miR-130b. The dual luciferase reporter assay, RT-PCR, Western blot and Immunofluorescence verified that miR-130b could directly bind to the 3′-UTR sequence of PTEN and suppress its expression in BC cells. Furthermore, the results showed that miR-130b-3p mimics promoted cell proliferation, migration and invasion as well as rearranged cytoskeleton in vitro, while proliferation was diminished when miR-130b-3p was restrained and cell apoptosis was also induced by anti-130b. Our research supplies the solid proof that miR-130b increases proliferation and metastasis by directly targeting PTEN on the base of integrating mRNA and miRNA expression profiles in BC. Chang et al. reported that MiR-130b increased proliferation and that Epithelial-Mesenchymal Transition (EMT)-induced metastasis through PTEN/p-AKT/HIF-1α signaling in hepatocellular carcinoma [26]. Yu et al. showed that MiR-130b plays an oncogenic role by inhibiting PTEN expression in esophageal squamous cell carcinoma cells [27]. Our experiment results supported these findings.

In the present research, we further demonstrated that miR-130b-3p might activate PI3K and integrin β1/FAK signaling pathways by targeting PTEN in bladder cancer cells. PTEN, a protein with homology to protein tyrosine phosphatases and tensin, is frequently deleted or mutated in various tumors, such as endometrium, breast, lung, kidney and bladder cancers as well as lymphoma [28,29]. It has been proposed that PTEN has double roles as a tumor suppressor: It may affect apoptosis and growth with its lipid phosphatase activity, which regulates levels of the PIP3 and Akt/PKB pathway, whereas it also participates in the regulation of cell migration, invasion and cytoskeletion by its protein tyrosine phosphatase activity targeting FAK and Shc [30]. PTEN is a negative regulator of the PI3K signaling pathway; inactivation of PTEN was identified as a trigger for progression from non-invasive to invasive bladder cancer. The integrated analysis of 131 muscle-invasive bladder cancer specimens revealed that dysregulation of the PI3K pathway was detected in 72% of the tumors; hence regulatory elements of the PI3K pathway have received much attention as potential therapeutic targets [31]. Our experiment results agree with these researches. β1 integrin is a kind of transmembrane receptor that communicates with a large number of downstream signaling molecules, including integrin-linked kinase (ILK), Caveolin-1, or FAK to trigger the survival pathway [32]. Therefore, targeting the miR-130b-3p could regulate multiple cancer-related signaling pathways, and provide the most effective therapeutic strategy for the bladder cancer treatment.

## 4. Materials and Methods

### 4.1. Patient Sample

Thirty pairs of bladder carcinoma and para-carcinoma tissues (2 cm away from the carcinoma lesion) from patients who had not undergone radiation therapy or chemotherapy in this study were from The First Affiliated Hospital of Chongqing Medical University. Informed consent was obtained from these patients. Samples were pathologically confirmed, and were rapidly put into liquid nitrogen after surgical operation. The research protocol (NO. 81572536, 26 February 2015) was authorized and supervised by The Ethics Committee of Chongqing Medical University. The investigations were carried out following the rules of the Declaration of Helsinki of 1975, revised in 2008.

### 4.2. RNA Extraction and Microarray Hybridization

RNA was utilized for microarray analysis from the four BC specimens and adjacent tissue specimens using TRIzol reagent (Takara, Dalian, China). The RNA quantification and quality assurance were confirmed by NanoDrop ND-1000 (OD 260 nm, NanoDrop, Wilmington, DE, USA), RNA Integrity and gDNA contamination were tested by denaturing agarose gel electrophoresis and estimated by the ratio of absorbance at 260 to 280 nm (A260/A280). The microarray hybridization was performed according to the manufacturer’s standard protocols (Agilent Technology, Shanghai, China) including purifying RNA, transcribing into fluorescent cDNA, and then hybridizing onto the Human lncRNA Array v3.0 (Arraystar, Shanghai, China) and the Human miRNA Array v2.0 (Arraystar). After the slides were baptised, the arrays were sweeped with an Agilent G2505C scanner (Agilent Technology).

### 4.3. Microarray Data Analysis

The captured array images were analyzed by Agilent Feature Extraction software (v11.0.1.1, Agilent Technology). Quantile normalization and subsequent data processing were executed with the Gene-Spring GX v11.5.1 software package (Agilent Technologies), R software package and gene expression dynamics inspector (GEDI). After quantile normalization of the raw data, miRNAs and mRNAs that were labeled as Present or Marginal (“All Targets Value”) in all four samples were selected for further data analysis. Statistically significant differential expressions of mRNAs and miRNAs between BC and paired non-tumor tissue were identified through hierarchical clustering. Significant differential expressed transcripts of mRNAs and miRNAs were respectively retained by screening fold change ≥2.0 and 1.5 as well as *p*-value < 0.05.

### 4.4. The Network of Target Gene Predictions

miRNA target prediction was performed using miRWalk2.0 (available on: http://mirwalk.uni-hd.de/) and miRanda (available on: http://www.microRNA.org) to identify potential paired miRNA/mRNA interaction via miRNA response elements. Furthermore, the Pearson correlation coefficient (PCC) between paired miRNA and mRNA according to their expression levels was calculated. The value of PCC ≤ −0.90, *p*-value < 0.01 was recommended. The qualified paired miRNA/mRNA interaction was drawn with the help of Cytoscape 3.01 (available on: http://apps.cytoscape.org).

### 4.5. qRT-PCR

qRT-PCR was conducted to confirm the microarray data from 30 BC patients. Reverse transcription PCR (RT-PCR) was performed on 500 ng of total RNA extracts that had been reverse transcribed into cDNA using PrimeScript Reverse Transcriptase (Takara, Dalian, China) according to the manufacturer’s guide. mRNA was transcribed into first-strand cDNA using a PrimeScript^®^ 1st Strand cDNA Synthesis Kit (Takara). By using SYBR Green chemistry (SYBR green II) (Takara), qRT-PCR was implemented with the ABI 7900HT sequence detection machine (Bio-Rad, Hercules, CA, USA). The specific primers were as follows: hsa-miR-199a-5p: F: 5′GGTGCCCAGTGTTCAGAC3′, R: 5′GTGCGTGTCGTGGAGTCG3′; hsa-miR-145-3p: F: 5′GGGGATTCCTGGAAATA3′, R: 5′GTGCGTGTCGTGGAGTCG3′; hsa-miR-106b-3p: F: 5′CCGCACTGTGGGTACT3′, R: 5′TGCGTGTCGTGGAGTC3′; hsa-miR-130b-3: F: 5′GGGCAGTGCAATGATGAAA3′, R: 5′GTGCGTGTCGTGGAGTCG3′; hsa-miR-99a-3p: F: 5′GGGCAAGCTCGCTTCTATG3′, R: 5′GTGCGTGTCGTGGAGTCG3′. PCR was executed in a 10 μL reaction volume and composed of an initial denaturation step at 95 °C for 3 min, followed by amplification with 40 cycles at 95 °C for 5 s and 60 °C for 30 s, then the melt curve step at 65 to 95 °C and the increment at 0.5 °C for 5 s. The threshold cycle (*C*t) was defined as the cycle number at which the fluorescence passed a predetermined threshold. The target and reference genes (U6) were amplified in separate wells in triplicate. Gene expressions were calculated with the comparative threshold cycle (2^-ΔΔ*C*t^) approach.

### 4.6. GO and KEGG Enrichment Analysis

The GO and KEGG enrichment analysis of co-expressing target genes were executed for the two most differentially up-regulated and three most differentially down-regulated miRNAs. Pathway analysis of lists of genes was achieved with the DAVID Functional Annotation Tool (available on: http://david.abcc.ncifcrf.gov/). Gene Ontology (GO) enrichment analysis was used to identify characteristic biological attributes of the co-expression target genes of differentially expressed miRNAs. GO-seq according to Wallenius non-central hyper-geometric distribution was performed for the GO enrichment analysis, which could adjust for bias of gene length.

### 4.7. Cells, Reagents and Antibodies

The bladder cancer cell lines EJ and T24 were obtained from the Chinese Science Institute (Shanghai, China). All the cells were grown in Roswell Park Memorial Institute (RPMI) 1640 medium (Gibico, Invitrogen, Carlsbad, CA, USA), supplemented with 10% fetal bovine serum (FBS) and 100 U/mL Penicillin and 0.1 mg/mL Streptomycin, then cultured at 37 °C and 5% CO_2_ atmosphere. Cells were transfected with the Lipofectamine 2000 (Invitrogen) following the manufacturer’s recommendations. Then, the cells were harvested at 48 h after transfection. The primary antibodies used were as follows: Rabbit–anti-human PTEN (Bioworld, Minneapolis, MN, USA, 1:500 for western blot (WB), 1:200 for Immunofluorescence (IF)), Rabbit–anti-human-ITGB1, PI3K protein (Cell Signaling Technology, Beverly, MA, USA, 1:1000 for WB, 1:500 for IF)—Rabbit–human-signalling pathway proteins were obtained from Bioworld Technology, Inc. (St. Louis, MO, USA, 1:500 for WB)—glyceraldehyde-3-phosphate dehydrogenase (GAPDH) Antibody (Bioworld, 1:2000). The sequences of human PTEN-3′-UTR were cloned into the pmiR-RB-Report™ vector (RIBOBIO, Guangzhou, China), and the corresponding mutant (Mut) 3′-UTR sequences were subsequently generated using overlap extension PCR and cloned into the pmiR-RB-Report™ vector. Hsa-miR-130b-3p mimics and the scramble control were produced by GenePharma (Shanghai, China). The micrOFF™ miRNA inhibitor (referred to as anti-130b) and anti-con were purchased from RIBOBIO. The micrOFF™ miRNA inhibitor is a specially modified chemical compound. The miRNA antagomir can bind to mature miRNA molecules to inhibit the effect of gene silencing. The secondary antibodies are from Beijing Zhongshan Biotechnology (Beijing, China).

### 4.8. Dual-Luciferase Reporter Assay

PTEN 3′-UTR was amplified by overlap extension PCR. The forward primer of wild 3′-UTR was 5′-CTCGAGATTTTTTTTTATCAAGAGGGA-3′. The reverse primer was 5′-ATTGCGGCCGCACAGCAGGTATTATGATTG-3′. The forward primer of mutated 3′-UTR was 5′-CTACCCCTAACGTGATGTGGCAACAGATAAGTT-3′; the reverse primer was 5′-TTGCCACATCACGTTAGGGGTAGGATGTGAACC-3′. Wild type and mutated 3′-UTRs were cloned into the pmiR-RB-Report™ vector (RIBOBIO).

The 293T cells at a density of 15,000 cells/well were put into a 96-well plate. The cells were co-transfected with 50 nM of miR-130b mimics or the scramble control and 100 ng/μL of pmiR-RB-REPORT^TM^-PTEN-UTR vector (RIBOBIO), respectively. The cell extracts were gathered 48 h after transfection. The luciferase activities were tested with the dual-luciferase reporter assay system (Promega Corp., Madison, WI, USA). The luciferase intensity was determined with a GLOMAX20/20 luminometer (Promega Corp) and normalized to the fireflyluciferase activity.

### 4.9. Cell Proliferation Assay

The cell proliferation rate was detected by CCK-8 assay. Cell Counting Kit-8 was bought from DingGuo (Beijing, China). Briefly, 2.0 × 10^3^ EJ/T24 cells were seed into 96-well plates for CCK-8. The fresh medium containing 10 µL CCK-8 was added to each well after 12, 24, 36, 48, and 72 h, respectively; the plates were shaken gently and then the cells were cultured at 37 °C for 2 h following the instructions. The absorbance was monitored with a plate reader at 490 nm. The assay was repeated three times. The Edu (5′-ethynyl-2′-deoxyuridine) analysis was executed with the Cell-Light™ Edu DNA Cell Proliferation Kit (#C10310, RIBOBIO) according to the manufacturer’s instructions. In the Edu assay, 1.0 × 10^5^ cells were put into 24-well plates, per well for 48 h, and then 50 μM of Edu was added to each well and cells were cultured for an additional 2 h at 37 °C. The cells were fixed with 4% formaldehyde for 30 min, added 2 mg/mL glycine for 5 min and treated with 0.5% Triton X-100 for 20 min at room temperature. After washing, the cells were incubated with Apollo reaction cocktail for 30 min at room temperature. Then, the cells were stained with Hoechst33342 for 30 min and visualized under a fluorescent microscope (Olympus Corporation, Tokyo, Japan). The Edu incorporation rate was expressed as the ratio of Edu positive cells (red cells) to total Hoechst33342 positive cells (blue cells). All experiments were done in triplicate.

### 4.10. Apoptosis Detection

TUNEL (TdT-UTP nick end labeling) analysis was executed by the one step TUNEL kit (#TUNEL-A2, Dingguo, Beijing, China). Concisely, the cells were fixed by 4% paraform, added 0.1% Triton X-100 for 3 min on ice, incubated with TUNEL for 60 min at 37 °C. The apoptotic cells revealed green fluorescence. The cell apoptosis rates were also detected with flow cytometry. Briefly, 1.0 × 10^5^ cultured cells were harvested by trypsinization, washed with phosphate buffer saline (PBS) and trypsinized into single cell suspensions. Then, the cells from each sample were stained with annexin V-FITC and propidium iodide (PI), and processed for Annexin V FITC/PI apoptosis detection (Becton Dickinson, New York, NY, USA) according to the manufacturer’s instructions. Annexin V-FITC cell apoptosis assay kit was purchased from Beyotime (#C1063, Shanghai, China). Experiments were carried out in triplicate.

### 4.11. Wound Healing and Cell Invasion Assay

The cells suspension (2.0 × 10^5^ cells/well) was plated into 6-well plates and grown to 80% confluence. Wounds were operated with 200 µL tip in the middle of the wells, and then the medium was changed with serum-free RPMI 1640 immediately. Pictures were taken using an inverted phase-contrast microscope (TE2000-U, NIKON, Tokyo, Japan) at 0 and 24 h. Wound healing was identified as the migration ability of cells. The width of wounds was detected in five independent wound sites per group. Cell invasion was performed with Matrigel Invasion chambers (BD Biosciences, New York, NY, USA). Matrigel (BD Biosciences) was mixed with precooled RPMI1640 without serum (1:8), then added into the upper chamber (8.0 μm membrane, Millpore). A total of 2 × 10^5^ resuspended cells in serum-free RPMI1640 were placed into the upper chamber, 800 µL of RPMI1640 containing 10% FBS was put into lower chamber. The cells were incubated for 12 h, the non-migrating cells in the upper chamber were softly erased with a cotton bud, the invading cells in the lower chamber were fixed and stained by crystal violet, then photographed and counted. All experiments were repeated three times.

### 4.12. Cytoskeleton

Cells were seeded on cover slips in 24-well plates for 48 h and rinsed three times using PBS, fixed by 4% paraform for 30 min, treated with 0.3% Triton X-100 for 5 min, then blocked with 3% BSA for 30 min at 37 °C. Next, cells were incubated with antibody against Paxillin (1:200 dilution) overnight at 4 °C. After, they were rinsed three times using PBS, followed by TRITC-conjugated secondary antibody for 1h, then treated with 1% phalloidine-FITC (Sigma Chemical Corp., La Jolla, CA, USA) for 40 min at 37 °C. After that, coverslips were incubated with 4’,6-diamidino-2-phenylindole (DAPI) for 10 min, and sealed with Antifade Solution. Then, the cells were scanned and photographed under Leica TCS-SP2 Laser Scanning Confocal Microscope (Leica, Wetzlar, Germany).

### 4.13. Western Blot Analysis

Concisely, total cellular protein lysates were sealed with Radio Immunoprecipitation Assay (RIPA) buffer containing proteinase inhibitor (Sigma Chemical Corp.). The 30 μg of protein was separated by 10% sodium dodecyl sulfate polyacrylamide gel electrophoresis (SDS PAGE) and transferred to polyvinylidene fluoride (PVDF) membranes. After blocking, the membranes were incubated overnight with primary antibodies (p-PI3K, p-AKT, p-GSK3β, PTEN, p-FAK and integrin β1, 1:500 dilution) at 4 °C. The membranes were incubated with goat anti-rabbit secondary IgG (1:2000 dilution) at 37 °C for 2 h and visualized with an enhanced chemiluminescence (ECL) detection system (Thermo Scientific, New York, NY, USA). Loading differences were normalized using a monoclonal GAPDH (1:2000) antibody.

### 4.14. HE, Immunohistochemistry and Immunofluorescence

Tissue samples of the 5-μm paraffin section were stained with HE for pathology observation. The sections were dewaxed and then submitted to a gradient of alcohol. They were treated with 0.3% TritonX-100 for 10 min and added with 0.3% H_2_O_2_ for 10 min, then blocked for 20 min. Sections were treated overnight in primary antibodies against PTEN and then treated with secondary antibodies (IgG/Bio) for 1 h, using SP (Streptavidin/Peroxidase) Histostain TM-Plus Kits, Diaminobenzidine (DAB) staining. The nuclei were counterstained by hematoxylin and then scanned with the microscope. For immunofluorescence, cells were placed on cover slips in 24-well plates for 24 h, fixed by paraform for 20 min, blocked for 30 min, and incubated overnight at 4 °C in primary antibodies against PTEN. The next day, sections were incubated with secondary antibodies (FITC-conjugated or TRITC-conjugated). Images were obtained using an Olympus multifunction microscope (Tokyo, Japan).

### 4.15. Statistical Methods

SPSS 18.0 and GraphPad Prism 5.0 statistical software (Graphpad Software Inc., San Diego, CA, USA) were utilized for the analysis with Student’s *t*-test. Results were expressed as the mean ± SD. *p*-Value < 0.05 was considered statistically significant.

## 5. Conclusions

Taken together, our results demonstrated that a genome-wide screen of miRNAs and targeting mRNAs revealed the negatively regulatory effect of miR-130b-3p on PTEN in bladder cancer by PI3K and integrin β1/FAK signaling pathways. Our findings suggest that the miR-130b could play a critical role in the progression and development of BC. Further research will be required to ascertain the molecular mechanism underlying miR-130b-3p in bladder cancer using a greater number of clinical samples with different pathologic classifications.

## Figures and Tables

**Table 1:** The 18 up-regulated miRNAs ranked by fold changes in microarray data.

UP		Fold Change	*p*-Value
ID	Name	Exp vs. Con	Exp vs. Con
145950	hsa-miR-33b-5p	3.336370154	0.004956079
146131	hsa-miR-2117	2.604240009	0.045920679
168915	hsa-miR-4780	2.543903736	0.042445646
17854	hsa-miR-106b-3p	2.535437308	0.034207452
10936	hsa-miR-130b-3p	2.276421905	0.047543441
42892	hsa-miR-450b-3p	1.995321427	0.023482703
10923	hsa-miR-107	1.989390422	0.006583535
17936	hsa-miR-16-1-3p	1.950937554	0.046962876
42730	hsa-miR-423-3p	1.839347459	0.018558695
42581	hsa-miR-513a-5p	1.795224692	0.044640818
148327	hsa-miR-3651	1.794198265	0.029542806
46869	hsa-miR-1258	1.723432724	0.020138235
169167	hsa-miR-4451	1.693943125	0.017475933
42949	hsa-miR-155-3p	1.692921273	0.048609104
17391	hsa-miR-635	1.626598085	0.002958794
13179	hsa-miR-455-5p	1.619979204	0.023576850
145745	hsa-miR-335-3p	1.597940804	0.041387192
145717	hsa-miR-516a/b-3p	1.579328007	0.044895980

Condition pairs: Experiment group vs. Control group (Exp vs. Con); Fold Change cut-off: 1.5; *p*-value cut-off: 0.05; Column “ID”: Array ID of the probes, each miRNA always has its unique probe, but some miRNAs may have two different probes; Column “Name”: The name of each miRNA; Column “Fold change”: The ratio of normalized intensities between two conditions (use normalized data, ratio scale); Column “*p*-value”: Paired *t*-test result between samples in different groups.

**Table 2:** The 81 down-regulated miRNAs ranked by fold changes in microarray data.

DOWN		Fold Change	*p*-Value
ID	Name	Exp vs. Con	Exp vs. Con
145651	hsa-miR-99a-3p	0.051086552	7.53934 × 10^5^
145943	hsa-miR-100-5p	0.067791739	0.002173279
30787	hsa-miR-125b-5p	0.084582742	0.005679508
42686	hsa-miR-136-3p	0.102904278	0.016379138
19591	hsa-miR-199b-5p	0.115965971	0.005383998
29562	hsa-miR-199a-5p	0.128659384	0.004991946
10995	hsa-miR-199a/b-3p	0.143691076	0.005761323
145822	hsa-miR-214-5p	0.195786477	0.000116725
42829	hsa-miR-127-3p	0.202381027	0.006742221
10943	hsa-miR-136-5p	0.219393229	0.004850101
31867	hsa-miR-145-3p	0.222153448	0.023978368
17944	hsa-miR-337-5p	0.238479942	0.012453995
46626	hsa-miR-30c-2-3p	0.240835077	0.001008995
11018	hsa-miR-218-5p	0.250517237	0.014066474
17482	hsa-miR-411-5p	0.251814653	0.016992894
169046	hsa-miR-4715-5p	0.259395379	0.013799259
33596	hsa-miR-126-5p	0.289376368	0.021690880
169415	hsa-miR-187-5p	0.293809285	0.004695797
146009	hsa-miR-376a-3p	0.296238972	0.006251505
42629	hsa-miR-376c-3p	0.298543576	0.024114100
11091	hsa-miR-377-3p	0.311789277	0.009371085
168980	hsa-miR-4324	0.313625447	0.023252715
145677	hsa-miR-139-5p	0.320773384	0.009280757
147673	hsa-miR-4262	0.361786249	0.012496365
148039	hsa-miR-3925-5p	0.363658051	0.021581694
148382	hsa-miR-3609	0.381682467	0.025550722
169383	hsa-miR-548ah-5p	0.436213703	0.001082679
42453	hsa-miR-376b/c-5p	0.448649921	0.002099911
168932	hsa-miR-5094	0.448868287	0.022425735
169149	hsa-miR-4659b-5p	0.455660642	0.002122712
148089	hsa-miR-208a-3p	0.459113699	0.007047771
148654	hsa-miR-184	0.475720561	0.023389351
11102	hsa-miR-410-3p	0.486892381	0.001975760
169038	hsa-miR-488-3p	0.496456609	0.004189093
42764	hsa-miR-412-3p	0.497046297	0.023298170
11104	hsa-miR-422a	0.507097110	0.021252147
147915	hsa-miR-3174	0.514617804	0.013270708
148187	hsa-miR-410-5p	0.521024486	0.024425287
13130	hsa-miR-517-5p	0.538378530	0.007681208
168818	hsa-miR-4774-5p	0.544885335	0.020040461
145640	hsa-miR-328-3p	0.548194103	0.009030944
147537	hsa-miR-4264	0.551794193	0.018874296
46623	hsa-miR-1273a	0.555475406	0.027998466
11111	hsa-miR-432-5p	0.564939359	0.022890766
145857	hsa-miR-154-5p	0.564947384	0.027411645
168683	hsa-miR-4792	0.569054172	0.027386896
146096	hsa-miR-764	0.595632201	0.025813179
168925	hsa-miR-1273g-3p	0.608040769	0.027206880
147776	hsa-miR-4317	0.610093911	0.026950961
42673	hsa-miR-337-3p	0.617048724	0.009417660
17958	hsa-miR-576-3p	0.638884891	0.012748315
148349	hsa-miR-3938	0.655804338	0.003662500
169369	hsa-miR-4490	0.658510235	0.009235676
147828	hsa-miR-3121-3p	0.661253760	0.011786378
42729	hsa-miR-34c-3p	0.665477769	0.013533460

## Supplementary Materials

Supplementary materials can be found at www.mdpi.com/1422-0067/18/1/78/s1.

## References

[1] Zhao F., Lin T., He W., Han J., Zhu D., Hu K., Li W., Zheng Z., Huang J., Xie W. (2015). Knockdown of a novel lincRNA AATBC suppresses proliferation and induces apoptosis in bladder cancer. Oncotarget.

[2] Zhuang J., Lu Q., Shen B., Huang X., Shen L., Zheng X., Huang R., Yan J., Guo H. (2015). TGFβ1 secreted by cancer-associated fibroblasts induces epithelial-mesenchymal transition of bladder cancercells through lncRNA-ZEB2NAT. Sci. Rep..

[3] Chatterjee A., Chattopadhyay D., Chakrabarti G. (2015). miR-16 targets Bcl-2 in paclitaxel-resistant lung cancer cells and overexpression of miR-16 along with miR-17 causes unprecedented sensitivity by simultaneously modulating autophagy and apoptosis. Cell Signal..

[4] Drusco A., Bottoni A., Laganà A., Acunzo M., Fassan M., Cascione L., Antenucci A., Kumchala P., Vicentini C., Gardiman M.P. (2015). A differentially expressed set of microRNAs in cerebro-spinal fluid (CSF) can diagnose CNS malignancies. Oncotarget.

[5] Frixa T., Donzelli S., Blandino G. (2015). Oncogenic microRNAs: Key players in malignant transformation. Cancers (Basel).

[6] Fernandez-Mercado M., Manterola L., Lawrie C.H. (2015). MicroRNAs in Lymphoma: Regulatory Role and Biomarker Potential. Curr. Genomics.

[7] Li Z., Li Y., Wang N., Yang L., Zhao W., Zeng X. (2016). miR-130b targets NKD2 and regulates the Wnt signaling to promote proliferation and inhibit apoptosis in osteosarcoma cells. Biochem. Biophys. Res. Commun..

[8] Egawa H., Jingushi K., Hirono T., Ueda Y., Kitae K., Nakata W., Fujita K., Uemura M., Nonomura N., Tsujikawa K. (2016). The miR-130 family promotes cell migration and invasion in bladder cancer through FAK and Akt phosphorylation by regulating PTEN. Sci. Rep..

[9] Li S., Geng J., Xu X., Huang X., Leng D., Jiang D., Liang J., Wang C., Jiang D., Dai H. (2016). miR-130b-3p Modulates epithelial-mesenchymal crosstalk in lung fibrosis by targeting IGF-1. PLoS ONE.

[10] Tu K., Liu Z., Yao B., Han S., Yang W. (2016). MicroRNA-519a promotes tumor growth by targeting PTEN/PI3K/AKT signaling in hepatocellular carcinoma. Int. J. Oncol..

[11] Jing X., Cheng W., Wang S., Li P., He L. (2016). Resveratrol induces cell cycle arrest in human gastric cancer MGC803 cells via the PTEN regulated PI3K/Akt signaling pathway. Oncol. Rep..

[12] Calderaro J., Rebouissou S., de-Koning L., Masmoudi A., Hérault A., Dubois T., Maille P., Soyeux P., Sibony M., de la Taille A. (2014). PI3K/AKT pathway activation in bladder carcinogenesis. Int. J. Cancer.

[13] Weinstein J.N., Akbani R., Broom B.M., Wang W., Verhaak R.G., McConkey D., Lerner S., Morgan M., Creighton C.J., Smith C. (2014). Comprehensive molecular characterization of urothelial bladder carcinoma. Nature.

[14] Zhang L.L., Liu J., Lei S., Zhang J., Zhou W., Yu H.G. (2014). PTEN inhibits the invasion and metastasis of gastric cancer via downregulation of FAK expression. Cell Signal..

[15] Pan J., Yang Q., Shao J., Zhang L., Ma J., Wang Y., Jiang B.H., Leng J., Bai X. (2016). Cyclooxygenase-2 induced β1-integrin expression in NSCLC and promoted cell invasion via the EP1/MAPK/E2F-1/FoxC2 signal pathway. Sci. Rep..

[16] Zhao X., He W., Li J., Huang S., Wan X., Luo H., Wu D. (2015). miRNA-125b inhibits proliferation and migration by targeting SphK1 in bladder cancer. Am. J. Transl. Res..

[17] Braicu C., Cojocneanu-Petric R., Chira S., Truta A., Floares A., Petrut B., Achimas-Cadariu P., Berindan-Neagoe I. (2015). Clinical and pathological implications of miRNA in bladder cancer. Int. J. Nanomed..

[18] Yao Y., Hu J., Shen Z., Yao R., Liu S., Li Y., Cong H., Wang X., Qiu W., Yue L. (2015). miR-200b expression in breast cancer: A prognostic marker and act on cell proliferation and apoptosis by targeting Sp1. J. Cell. Mol. Med..

[19] Gao J., Zhu J., Li H.Y., Pan X.Y., Jiang R., Chen J.X. (2011). Small interfering RNA targeting integrin-linked kinase inhibited the growth and induced apoptosis in human bladder cancer cells. Int. J. Biochem. Cell. Biol..

[20] Zeng L.P., Hu Z.M., Li K., Xia K. (2016). miR-222 attenuates cisplatin-induced cell death by targeting the PPP2R2A/Akt/mTOR axis inbladder cancer cells. J. Cell. Mol. Med..

[21] Geng J., Fan J., Ou-yang Q., Zhang X., Zhang X., Yu J., Xu Z., Li Q., Yao X., Liu X. (2014). Loss of PPM1A expression enhances invasion and the epithelial-to-mesenchymal transition inbladder cancer by activating the TGF-β/Smad signaling pathway. Oncotarget.

[22] Chen M.F., Zeng F., Qi L., Zu X.B., Wang J., Liu L.F., Li Y. (2014). Transforming growth factor-β1 induces epithelial-mesenchymal transition and increased expression of matrix metalloproteinase-16 via miR-200b downregulation in bladder cancercells. Mol. Med. Rep..

[23] Lai K.W., Koh K.X., Loh M., Tada K., Subramaniam M.M., Lim X.Y., Vaithilingam A., Salto-Tellez M., Iacopetta B., Ito Y. (2010). MicroRNA-130b regulates the tumour suppressor RUNX3 in gastric cancer. Eur. J. Cancer.

[24] Wu X., Weng L., Li X., Guo C., Pal S.K., Jin J.M., Li Y., Nelson R.A., Mu B., Onami S.H. (2012). Identification of a 4-microRNA signature for clear cell renal cell carcinoma metastasis and prognosis. PLoS ONE.

[25] Li B.L., Lu C., Lu W., Yang T.T., Qu J., Hong X., Wan X.P. (2013). miR-130b is an EMT-related microRNA that targets DICER1 for aggression in endometrial cancer. Med. Oncol..

[26] Chang R.M., Xu J.F., Fang F., Yang H., Yang L.Y. (2016). MicroRNA-130b promotes proliferation and EMT-induced metastasis via PTEN/p-AKT/HIF-1α signaling. Tumour Biol..

[27] Yu T., Cao R., Li S., Fu M., Ren L., Chen W., Zhu H., Zhan Q., Shi R. (2015). miR-130b plays an oncogenic role by repressing PTEN expression in esophageal squamous cell carcinoma cells. BMC Cancer.

[28] Tang C., Guo J., Chen H., Yao C.J., Zhuang D.X., Wang Y., Tang W.J., Ren G., Yao Y., Wu J.S. (2015). Gene mutation profiling of primary glioblastoma through multiple tumor biopsy guided by ^1^H-magnetic resonance spectroscopy. Int. J. Clin. Exp. Pathol..

[29] Yu T., Liu L., Li J., Yan M., Lin H., Liu Y., Chu D., Tu H., Gu A., Yao M. (2015). miRNA-10a is upregulated in NSCLC and may promote cancer by targeting PTEN. Oncotarget.

[30] Villa-Moruzzi E. (2013). PTPN12 controls PTEN and the AKT signalling to FAK and HER2 in migrating ovarian cancer cells. Mol. Cell. Biochem..

[31] Carneiro B.A., Meeks J.J., Kuzel T.M., Scaranti M., Abdulkadir S.A., Giles F.J. (2015). Emerging therapeutic targets in bladder cancer. Cancer Treat. Rev..

[32] Luo C.W., Wu C.C., Ch’ang H.J. (2014). Radiation sensitization of tumor cells induced by shear stress: The roles of integrins and FAK. Biochim. Biophys. Acta.

