# Supplementary material for: Genome-Wide Screen of miRNAs and Targeting mRNAs Reveals the Negatively Regulatory Effect of miR-130b-3p on PTEN by PI3K and Integrin β1 Signaling Pathways in Bladder Carcinoma
Source: Int J Mol Sci. 2016 Dec 31;18(1):78. doi: 10.3390/ijms18010078 (PMC5297712; doi:10.3390/ijms18010078)
Supplement: Supplementary file 1 [file ijms-18-00078-s001.pdf]

Supplementary Materials: Genome-Wide Screen of miRNAs and Targeting mRNAs Reveals the Negatively Regulatory Effect of miR-130b-3p on PTEN by PI3K and Integrin β1 Signaling Pathways in Bladder Carcinoma

Mengxin Lv, Zhenyu Zhong, Hong Chi, Mengge Huang, Rong Jiang and Junxia Chen

Table S1. Clinical pathologic characteristics of study subjects.

Characteristic		Number of Patients
All patients		30 (100%)
Gender	Male	21 (70%)
	Female	9 (30%)
Age at diagnosis	<70 years	24 (80%)
	≥70 years	6 (20%)
Pathologic grade	Low grade	8 (26.7%)
	High grade	22 (73.3%)
TNM stage	Ta	1 (3.3%)
	T1	5 (16.7%)
	≥T2	24 (80%)
	N0	23 (76.7%)
	≥N1	7 (23.3%)
	M0	29 (96.7%)
	M1	1 (3.3%)

TNM: Tumor Node Metastasis.
